# IV injection of polystyrene beads for mouse model of sepsis causes severe glomerular injury

**DOI:** 10.1186/2052-0492-2-21

**Published:** 2014-03-06

**Authors:** Hajime Arima, Hiroyuki Hirate, Takeshi Sugiura, Shugo Suzuki, Satoru Takahashi, Kazuya Sobue

**Affiliations:** Department of Anesthesiology and Medical Crisis Management, Nagoya City University Graduate School of Medical Sciences, 1 Kawasumi, Mizuho-cho, Mizuho-ku, Nagoya, 467-8601 Japan; Department of Experimental Pathology and Tumor Biology, Nagoya City University Graduate School of Medical Sciences, 1 Kawasumi, Mizuho-cho, Mizuho-ku, Nagoya, 467-8601 Japan

**Keywords:** IV injection, Polystyrene beads, Glomerular injury

## Abstract

**Background:**

Infusion fluids may be contaminated with different types of particulates that are a potential health hazard. Particulates larger than microvessels may cause an embolism by mechanical blockage and inflammation; however, it has been reported that particulates smaller than capillary diameter are relatively safe. Against such a background, one report showed that polystyrene beads smaller than capillary diameter decreased tissue perfusion in ischemia–reperfusion injury. This report suggested that polystyrene beads from 1.5- to 6-μm diameter (dia.) may have unfavorable effects after pretreatment.

Here, we investigated whether injection of polystyrene beads (3- and 6-μm dia.) as an artificial contaminant of intravenous fluid after lipopolysaccharide (LPS) injection affected mortality and organ damage in mice.

**Methods:**

Mice were divided into four groups and injected: polystyrene beads only, LPS only, polystyrene beads 30 min after LPS, or saline. A survival study, histology, blood examination, and urine examination were performed.

**Results:**

The survival rate after LPS and polystyrene bead (6-μm dia.) injection was significantly lower than that of the other three groups. In the kidney sections, injured glomeruli were significantly higher with LPS and polystyrene bead injection than that of the other three groups. LPS and polystyrene bead injection decreased the glomerular filtration rate and led to renal failure. Inflammatory reactions induced with LPS were not significantly different between with or without polystyrene beads. Polystyrene beads were found in urine after LPS and polystyrene bead injection.

**Conclusions:**

Injection of polystyrene beads after LPS injection enhanced glomerular structural injury and caused renal function injury in a mouse sepsis model.

## Background

Infusion fluids may be contaminated with particulates, bacteria, endotoxins, precipitates, large lipids, and air. Different types of particulates have been described and recognized as a potential health hazard [1–3]. Particulates larger than microvessels may cause an embolism by mechanical blockage. In addition, larger particulates activate platelets and neutrophils and have indirect effects on vasomotor activity, including effects on the microcirculation, causing thrombi and granuloma formation [4–6]; however, there are no established animal models showing that larger particulates cause significant systemic functional or morphological injuries [4, 7] but only local injuries such as the formation of foreign body granulomas in the lungs and other organs. The reason for the lack of animal models is unknown, but in previous studies, larger particulates have been injected into healthy animals. In addition, the injection of particulates smaller than the inner luminal diameter of microvessels is relatively safe [8]. Smaller particulates are rapidly eliminated from the blood stream within the first 60–90 min after injection. These particulates mainly accumulate in the liver.

Against such a background, one report showed that polystyrene beads as an artificial intravenous fluid contaminant smaller than the inner luminal diameter of microvessels decreased microcirculatory tissue perfusion in ischemia–reperfusion injury [4]. In this report, injection of polystyrene beads (1.5-, 3-, 4.5-, and 6-μm diameter (dia.)) did not decrease capillary perfusion, but injection of polystyrene beads 10-μm diameter or larger resulted in a statistically significant loss of capillary perfusion in non-ischemic muscle tissue. On the other hand, even the smallest 1.5-μm diameter polystyrene beads significantly reduced capillary perfusion from the baseline value in post-ischemic muscle tissue. This report suggested that polystyrene beads smaller than 6-μm diameter do not cause embolisms, but this size of polystyrene bead may have unfavorable effects after pretreatment.

We therefore hypothesized that smaller particulates that do not cause embolisms have unfavorable effects on septic shock animals with the involvement of lipopolysaccharide (LPS). Here, we used polystyrene beads as an artificial intravenous fluid contaminant and investigated the effect of 3- and 6-μm dia. polystyrene bead injection after LPS injection in mice. First, mortality was investigated and then organ damage was screened by histology.

## Methods

### Animal protocols

After institutional ethics approval (conforming to the guidelines of the Centre for Animal Science, Nagoya City University Graduate School of Medical Sciences), male C57 BL6 non-SPF mice (6–7 weeks old, 20–25 g) obtained from Japan SLC, Inc. (Hamamatsu, Japan) were used in all experiments and were housed at 22–24°C under a 12-h light/12-h dark cycle.

Animals were randomized to polystyrene bead injection (10^6^–10^9^/kg, 3- or 6-μm dia., Polybead Microspheres 3.00 or 6.00 μm; Polysciences, Inc., Warrington, PA, USA. polystyrene bead-only group; Bead group), LPS injection (5 mg/kg; Sigma-Aldrich Co., St. Louis, MO: LPS-only group; LPS group), polystyrene bead injection 30 min after LPS injection (L + B group), or saline injection (5 ml/kg, Control group). The size of polystyrene beads used does not decrease capillary perfusion [4]. The dose of polystyrene beads used was not high compared with another report [8]. It has been reported that 8.3 × 10^8^/kg polystyrene beads (4.5-μm dia.) did not cause any macroscopic, clinicopathological, or histopathological changes in rats [8].

Under inhaled anesthesia with 1.5%–2% isoflurane, the fur on the right-hand side of the neck of the mouse was shaved and the skin was disinfected using an iodine solution. A longitudinal incision of about 15 mm was made in the neck of the animal, just inside the right front leg. After careful removal of the connective tissue surrounding the jugular vein, the indicated drug was slowly (over 10 s) injected through the jugular vein using a 27-gauge needle, and the skin was sutured [9]. Drugs were diluted with saline, and the injection volume was adjusted to about 0.1 ml. During the surgical procedure, the body temperature of the animals was maintained using an electrical heating pad. After recovery from anesthesia, the animals were observed and housed individually in cages.

### Survival study

After injection of the indicated drug, the animals were housed individually in cages. A status of dead or alive was recorded every 24 h for 10 days.

### Histology

At the indicated time after the injection of the indicated drug, the animals were euthanized, and the lung, liver, kidney, and spleen were removed and fixed in 10% buffered formalin. Sections of the lung, liver, and spleen were processed for hematoxylin and eosin (HE) staining. Sections of the kidney were processed for HE staining and periodic acid methenamine silver (PAM) staining. The histological samples were evaluated in a blinded fashion by only one pathologist who was not aware of the randomization of animals. For evaluation of glomeruli in each kidney section, the severity of the injury was not taken into account. Glomeruli with thickened and/or degenerated basement membrane detected by PAM staining were defined as ‘injured glomeruli’.

### Sample collection for blood examination

At the indicated time after injection of the indicated drug, the animals were euthanized and arterial blood samples were collected. Serum blood urea nitrogen (BUN) and creatinine were measured by an automated clinical chemistry analyzer (System 7700; Hitachi High-Technologies Corporation, Tokyo, Japan) with the UV kinetic method for BUN and enzymatic methods for creatinine. Blood counts and differential counts of leukocytes were measured by an automated blood count analyzer (XE-2100; SYSMEX Co., Kobe, Japan) with the flow cytometry method. Concentrations of interleukin (IL)-6 and cystatin C in serum were measured in duplicates using a commercial kit (Quantikine ELISA; R&D Systems, Inc., Minneapolis, MN, USA).

### Detection of polystyrene beads in glomeruli of kidneys

Dyed polystyrene beads (10^8^/kg, 6-μm dia., Polybead Polystyrene Yellow Dyed Microspheres 6.00 μm, Polysciences, Inc.) were used for ease of identification. Animals were randomized to dyed polystyrene bead injection (10^8^/kg) (Dyed Bead group) or dyed polystyrene bead injection (10^8^/kg) 30 min after LPS injection (5 mg/kg, L + DB group). At the indicated time after injection of the indicated drug, the animals were euthanized, the kidneys were removed and embedded in Tissue-Tek O.C.T. Compound (Sakura Finetek Japan Co., Ltd., Tokyo, Japan) in cryomolds, and then frozen in liquid nitrogen. Twenty micron, frozen serial sections cut on a standard cryostat were mounted, fixed in formalin, stained with HE, and rinsed in distilled water. Because polystyrene beads are soluble in xylene, slides were analyzed under water-soluble conditions. The histological samples were evaluated in a blinded fashion by only one pathologist who was not aware of the randomization of animals.

### Detection of polystyrene beads in urine

Dyed polystyrene beads of 6-μm diameter were used for ease of identification. After injection of the indicated drug, the animals were placed individually in metabolism cages for urine collection. After urine collection, they were microscopically evaluated for the presence of beads, which were counted.

### Statistical analysis

Data are presented as the mean ± SE. For survival analysis, the Kaplan–Meier method was used. For multiple comparisons, one-way analysis of variance followed by Tukey's multiple comparison test or two-way analysis of variance followed by the Bonferroni multiple comparison test was used. *P* < 0.05 was considered significant. Analysis was performed with GraphPad Prism 4 (GraphPad Software, Inc., La Jolla, CA, USA).

## Results

### Survival rate

**Figure 1:**
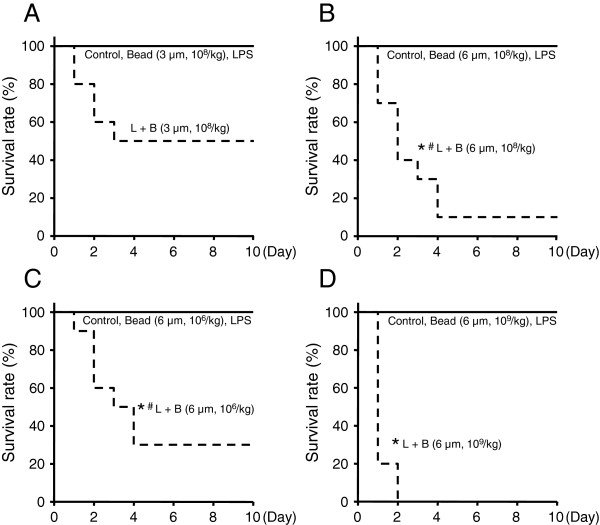
**Survival study.** Both 3- and 6-μm diameter polystyrene beads were investigated. Mice were randomized to polystyrene bead injection (10^6^, 10^8^, or 10^9^/kg) (Bead), LPS injection (5 mg/kg) (LPS), polystyrene bead injection (10^6^, 10^8^, or 10^9^/kg) 30 min after LPS injection (5 mg/kg) (L + B), or saline injection (5 ml/kg) (Control) and treated as described. The animals were observed in cages, and the status of dead or alive was recorded every 24 h for 10 days. *n* = 10 in each group. Asterisk, significantly different from Control at *P* < 0.05. Number sign, significantly different from 10^9^/kg within the L + B groups of 6-μm diameter polystyrene beads. **(A)** Survival curve of study (polystyrene beads 3-μm diameter, 10^8^/kg). **(B)** Survival curve of study (polystyrene beads 6-μm diameter, 10^8^/kg). **(C)** Survival curve of study (polystyrene beads 6-μm diameter, 10^6^/kg). **(D)** Survival curve of study (polystyrene beads 6-μm diameter, 10^9^/kg).

First, polystyrene beads of 3- and 6-μm diameter (10^8^/kg) were tested. After injection, the animals were followed for 10 days. In the Bead group, LPS group, and Control group with polystyrene beads of 3- and 6-μm diameter, no mice died during the 10 days. In the L + B group with 3-μm diameter beads, half of the mice died within 3 days, but the rest of the mice did not die during the 10 days (Figure 1A). This survival rate of the L + B group (3-μm dia., 10^8^/kg) was not significantly lower than that of the Control group. In the L + B group with 6-μm diameter beads, nine mice died within 4 days, but one mouse did not die during the 10 days (Figure 1B). This survival rate of the L + B group (6-μm dia., 10^8^/kg) was significantly lower than that of the Control group. Next, 10^6^ and 10^9^/kg of beads (6-μm dia.) were tested. The survival rates of the L + B groups (6-μm dia., 10^6^ and 10^9^/kg) were significantly lower than that of the Control group (Figure 1C,D). Compared with the survival rates within the L + B groups of 6-μm dia., 10^9^/kg is significantly lower than 10^8^ and 10^6^/kg. The survival rates of 10^8^/kg (6-μm dia., L + B group) was lower than 10^6^/kg (6-μm dia., L + B group), but the difference was not significant. Beads after LPS injection decreased the survival rate in a dose-dependent manner.

### Histology

From the survival study, it became clear that polystyrene beads of 6-μm diameter have significantly harmful effects. Therefore, polystyrene beads of 6-μm diameter and 10^8^/kg were used for the following experiments. To determine which organ was damaged, the lung, liver, kidney, and spleen were examined histologically. There was no significant difference in the lung sections from the four groups (data not shown).

**Figure 2:**
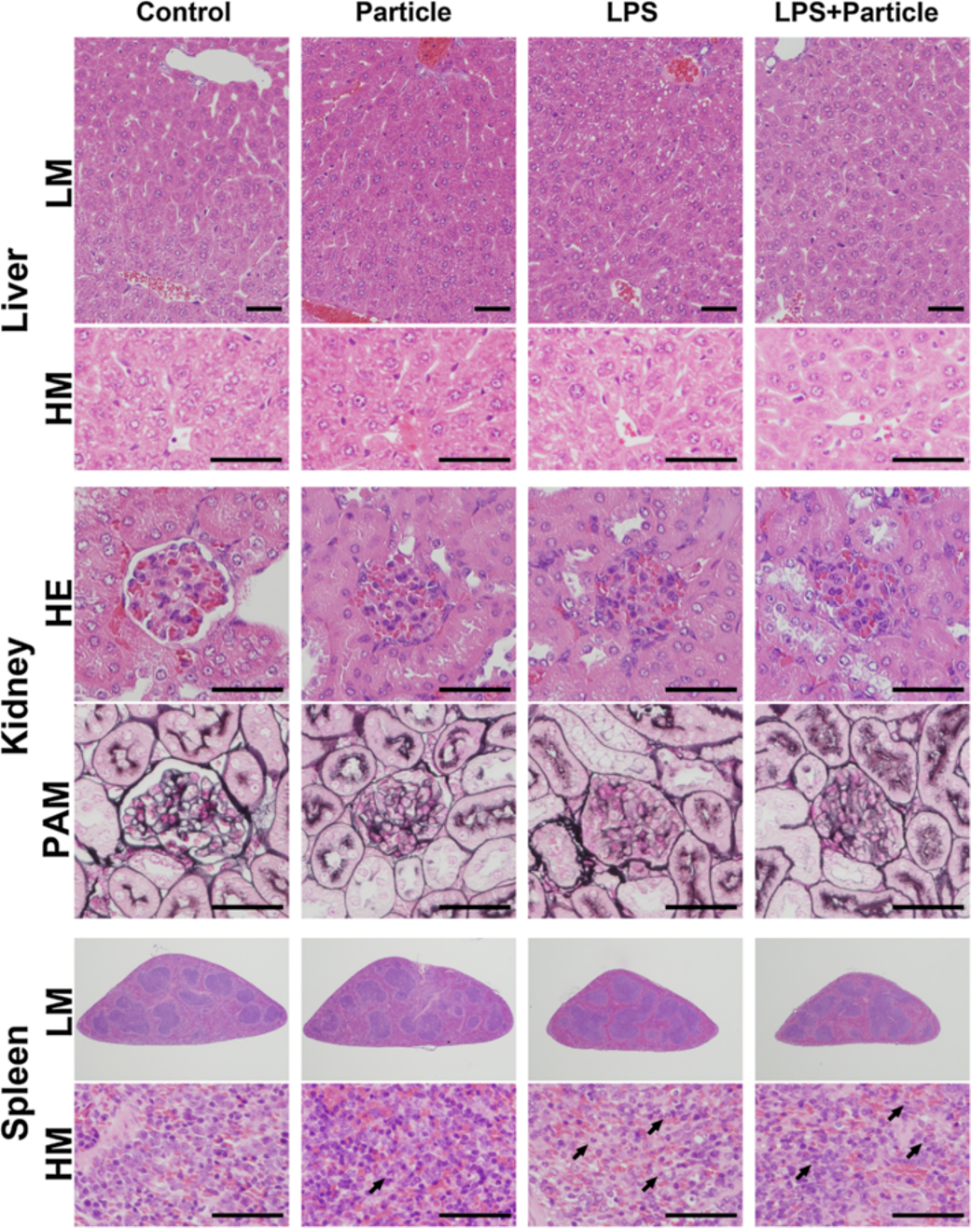
**Histology.** Mice were randomized to polystyrene bead injection (10^8^/kg, 6-μm dia.) (Bead), LPS injection (5 mg/kg) (LPS), polystyrene bead injection (10^8^/kg, 6-μm dia.) 30 min after LPS injection (5 mg/kg) (L + B), or saline injection (5 ml/kg) (Control) and treated as described. Forty-eight hours after injection, the liver, kidney, and spleen were removed. Sections of the liver and spleen were processed for hematoxylin and eosin (HE) staining. Sections of each kidney tissue were processed for HE staining and periodic acid methenamine silver (PAM) staining. Horizontal line is HE staining of liver (low and high magnification), HE stain of kidney, PAM staining of kidney, and HE staining of spleen (low and high magnification), respectively. Vertical lines are Control, Bead, LPS, and L + B, respectively. Arrows indicate neutrophils. Control (*n* = 4), Bead (*n* = 4), LPS (*n* = 4), L + B (*n* = 5). LM, low magnification; HM, high magnification; Black bar, 100 μm.

In the liver sections (Figure 2, upper and second column), there were granules of glycogen in the liver cells around central venous lesions in the Control group and Bead group. Meanwhile, granules of glycogen in the liver cells involving the entire liver lobule were detected in the LPS group and L + B group. In the L + B group, two fifth of the mice had fewer granules of glycogen in the liver cells. Hepatic lipidosis around portal lesions was also found in some of the LPS group and L + B group. There was no necrosis in any group. The number of Kupffer cells was not different among the groups (data not shown). The changes in the liver might have been toxic reactions to LPS but not to particles.

**Figure 3:**
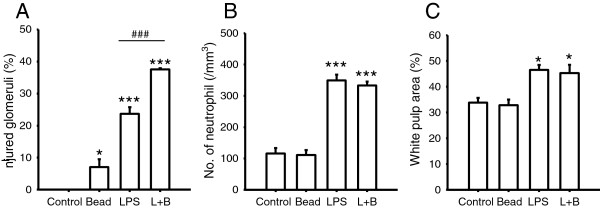
**Analysis of histology.** In the sections obtained from Figure 2, the percentage of injured glomeruli in the kidney, infiltration of neutrophils into red pulp per unit area, and percentage of white pulp area of the spleen were analyzed. Control (*n* = 4), Bead (*n* = 4), LPS (*n* = 4), L + B (*n* = 5). Single asterisk, triple asterisks, significantly different from Control at *P* < 0.05, 0.001, respectively. Triple number signs, significantly different between LPS and L + B at *P* < 0.001. **(A)** Percentage of injured glomeruli in the kidney. Injured and uninjured glomeruli in each kidney section of Figure 1 were counted, and the percentages of injured glomeruli are presented. **(B)** Infiltration of neutrophils into red pulp per unit area. In spleen sections of Figure 1, neutrophils in red pulp were counted and presented as per unit area. **(C)** Percentage of white pulp area of spleen. In the spleen sections of Figure 1, areas of white and red were measured, and the percentage of the white area is presented.

In kidney sections (Figure 2, third and fourth columns), there was a very slight effect when treated with polystyrene beads only. The thickness of the glomerular basement membrane in a small number of glomeruli was slightly increased by polystyrene beads that were counted as ‘injured glomeruli’. In the LPS group and L + B group, the basement membrane of some glomeruli was thickened and/or degenerated, which was defined as ‘injured glomeruli’. Slight mesangial proliferation was also detected in the LPS group and L + B group. No extracapillary proliferation or sclerosis in glomeruli was detected in any groups. The percentage of injured glomeruli is presented in Figure 3A. Injured glomeruli were significantly increased by treatment with LPS with or without polystyrene beads and were significantly higher in the L + B group than LPS group. There was no tubular damage or necrosis in any group. This means that glomeruli were injured by LPS. Polystyrene beads had only small effects on glomeruli but had synergetic effects with LPS injection.

In the spleen sections (Figure 2, fifth and lower column), infiltration of neutrophils was detected in the red pulp of the spleen. Neutrophils in the red pulp per unit area were significantly increased by LPS treatment, but not by particle treatment (Figure 3B). The percentage of white pulp area was significantly increased by LPS treatment (Figure 3C). There was no significant difference between the LPS group and L + B group in the infiltration of neutrophils and percentage of white pulp area. This means that polystyrene beads did not affect systemic inflammatory reactions induced by LPS.

### Biochemical and hematological examinations

**Figure 4:**
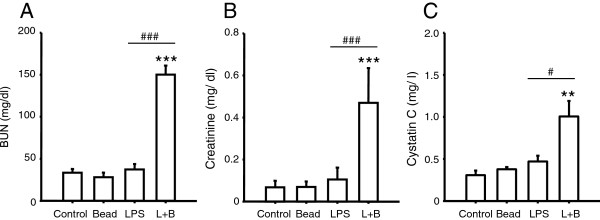
**Evaluation of kidney function.** Mice were randomized to polystyrene bead injection (10^8^/kg, 6-μm dia.) (Bead), LPS injection (5 mg/kg) (LPS), polystyrene bead injection (10^8^/kg, 6-μm dia.) 30 min after LPS injection (5 mg/kg) (L + B), or saline injection (5 ml/kg) (Control) and treated as described. Forty-eight hours after injection, serum was obtained and measured. *n* = 4 in each group. Double asterisks, triple asterisks, significantly different from Control at *P* < 0.01, 0.001, respectively. Single number sign, triple number signs, significantly different between LPS and L + B at *P* < 0.05, 0.001, respectively. **(A)** Serum BUN 48 h after injection. **(B)** Serum creatinine 48 h after injection. **(C)** Serum cystatin C 48 h after injection.

From the histological study, it became clear that the kidney was a key organ. Thus, to evaluate kidney function, serum BUN, creatinine, and cystatin C were measured. BUN, creatinine, and cystatin C were significantly increased only in the L + B group (Figure 4A,B,C). This means that saline, polystyrene beads only, and LPS only did not cause acute kidney injury; however, injection of polystyrene beads 30 min after LPS injection decreased the glomerular filtration rate and led to renal failure [10, 11].

**Figure 5:**
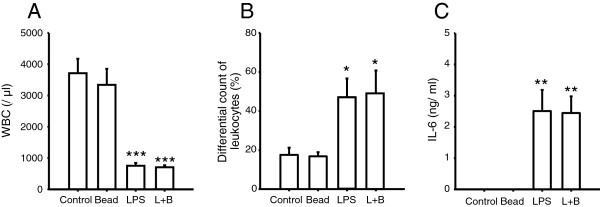
**Evaluation of inflammatory reaction.** Mice were randomized to polystyrene bead injection (10^8^/kg, 6-μm dia.) (Bead), LPS injection (5 mg/kg) (LPS), polystyrene bead injection (10^8^/kg, 6-μm dia.) 30 min after LPS injection (5 mg/kg) (L + B), or saline injection (5 ml/kg) (Control) and treated as described. Twenty-four or 48 h after injection, serum was obtained and measured. *n* = 4 in each group. Single asterisk, double asterisks, third asterisks, significantly different from Control at *P* < 0.05, 0.01, 0.001, respectively. **(A)** White blood cell count 48 h after injection. **(B)** Differential count of leukocytes 48 h after injection. **(C)** Serum IL-6 24 h after injection.

Findings from the histological study showed that polystyrene beads did not affect systemic inflammatory reactions induced by LPS. To evaluate the inflammatory reaction in blood, the blood count, differential count of leukocytes, and serum IL-6 were measured. White blood cell count was significantly decreased by treatment with LPS with or without polystyrene beads, but there was no difference between the LPS group and L + B group (Figure 5A). Differential count of leukocytes and serum IL-6 were significantly increased by treatment with LPS with or without polystyrene beads, but there was no significant difference between the LPS group and L + B group (Figure 5B,C). Serum IL-6 of LPS group and L + B group returned to the baseline level 48 h after injection (data not shown). This means that polystyrene beads did not affect systemic inflammatory reactions induced by LPS [12].

### Examination of dyed polystyrene beads in glomerulus and urine

**Figure 6:**
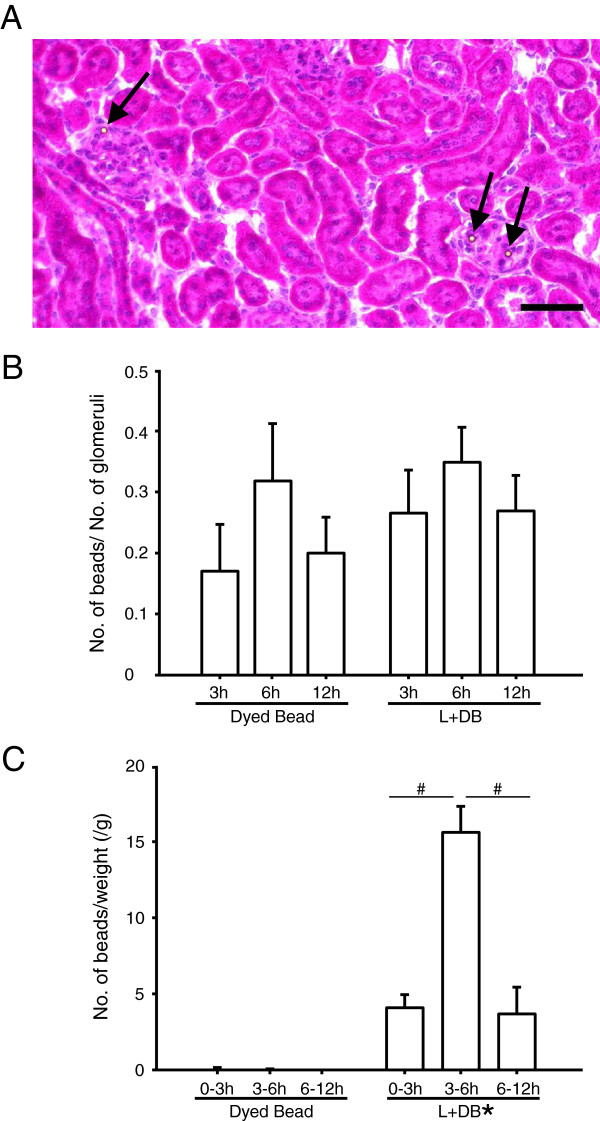
**Detection of polystyrene beads in glomeruli of kidneys and urine.** Mice were randomized to dyed polystyrene bead injection (10^8^/kg, 6-μm dia.) (Dyed Bead) or dyed polystyrene bead injection (10^8^/kg, 6-μm dia.) 30 min after LPS injection (5 mg/kg) (L + DB) and treated as described. **(A)** Three, six, or twelve hours after injection, the kidney sections were obtained. Dyed Bead (*n* = 3), L + DB (*n* = 5). Picture shows one of the Sections 6 h after injection in L + DB. Dyed polystyrene beads are found in the glomeruli (arrows). Black bar, 100 μm. **(B)** In the sections obtained from Figure 6A, dyed polystyrene beads in the glomeruli were counted and presented per number of glomeruli. Dyed Bead (*n* = 3), L + DB (*n* = 5). **(C)** Mice were randomized to Dyed Bead or L + DB and treated as described. Urine 0–3, 3–6, and 6–12 h after injection was collected, and dyed polystyrene beads were counted and presented per weight of animals. Dyed Bead (*n* = 5), L + DB (*n* = 5). Asterisk, significantly different between Dyed Bead and L + DB at *P* < 0.0001. Number sign, significantly different from 3–6 h after injection of L + DB at *P* < 0.001.

We could not detect polystyrene beads by common HE staining in the kidney. Polystyrene beads are dissolved by organic solvents, which are common drugs for histology. We therefore tried frozen section techniques without organic solvents to investigate the mechanism of glomerular injury associated with polystyrene beads and LPS. In addition, dyed polystyrene beads were injected instead of normal polystyrene beads to make them easy to find under a microscope. In both the Dyed Bead group and L + DB group, dyed polystyrene beads were detected in glomeruli (Figure 6A). Dyed polystyrene beads in glomeruli were counted but there was no significant difference between the Dyed Bead group and L + DB group (Figure 6B).

Next, dyed polystyrene beads were counted in urine (Figure 6C). There was a significant difference between the Dyed Bead group and L + DB group. Few dyed polystyrene beads were found in urine in the Dyed Bead group. Dyed polystyrene beads were eliminated in urine from 0 to 12 h after injection in the L + DB group. The elimination of dyed polystyrene beads peaked from 3 to 6 h after injection. These studies using dyed polystyrene beads revealed that injected polystyrene beads stuck in the glomeruli in the Dyed Bead group and L + DB group, whereas polystyrene beads passed through the injured glomeruli only in the L + DB group.

## Discussion

Polystyrene bead injection alone had no effect on the survival rate, kidney function, or inflammatory reaction but slightly increased the thickness of the glomerular basement membrane. LPS injection alone also had no effect on the survival rate or kidney function, but evoked toxic and inflammatory reactions and injured glomeruli. Polystyrene bead injection 30 min after LPS injection had the worst effect on the survival rate; however, the systemic inflammatory reaction associated with LPS was the same with or without polystyrene beads. A difference between with and without polystyrene beads after LPS injection was found in the kidney. Polystyrene bead injection after LPS injection severely damaged the glomerulus. It can be summarized that polystyrene beads damaged the glomerular basement membrane only slightly but did not affect inflammation reactions induced by LPS. Once the glomerulus had been damaged by LPS, polystyrene beads accelerated the damage, decreased the glomerular filtration rate, and led to renal failure.

The details of glomerular injury due to LPS and polystyrene beads are not clear. It is said that LPS induces glomerular injury. The renal filtration barrier consists of fenestrated endothelial cells, the glomerular basement membrane, and podocytes of epithelial cells. Foot processes of podocytes interdigitate with one another and form a slit diaphragm. Nephrin is a transmembrane protein that is a key structural component of the slit diaphragm and is located at the slit diaphragm between adjacent podocyte foot processes [13]. LPS is known to damage glomerular nephrin. This damage led to breakdown of the slit diaphragm and renal filtration barrier and then induced albuminuria in mice [14–17]. In our study, polystyrene beads stuck in the glomerulus were not significantly different with or without LPS; however, polystyrene beads passed through the renal filtration barrier injured by LPS. It is suggested that local mechanical injury due to polystyrene beads is one of the causes of glomerular damage. Polystyrene beads may mechanically injure the renal filtration barrier injured by LPS as they attach and pass through the barrier. Due to the combination of these two types of damage, from LPS and polystyrene beads, the glomerulus might be severely injured. Our results that polystyrene beads did not affect systemic inflammation reactions induced by LPS, larger polystyrene beads of 6-μm diameter were more harmful than smaller beads of 3-μm diameter and polystyrene beads of 6-μm diameter decreased the survival rate in a dose-dependent manner, support our hypothesis.

It is not clear whether our model of polystyrene beads and LPS is relevant to clinical sepsis. There are two limitations of our study. The first limitation is artificial intravenous fluid contaminants. In the clinical setting, many particulates have been found in used in-line IV filters [18]. The amount was a mean of 550 particulates/cm^2^ (8–1,993 particulates/cm^2^). Particulate size ranged from 5 to >100 μm with the majority between 5 and 50 μm. Silicon, which originates mainly from storage in glass ampules, was the most frequently detected element in the analysis of individual particulates. It has been reported that glass particulates had immunomodulating effects on endothelial cells and macrophages *in vitro*[18]. This study used inhomogeneous glass particulates (2–20 μm). Glass particulates have an angular shape and crystalline appearance. Polystyrene beads are different from the observed particulates. In the experimental setting, similarly sized glass particulates are not in commercial production and are therefore unavailable, so, we used polystyrene beads as an artificial intravenous fluid contaminant, 3 and 6 μm in diameter and spherical. The second limitation is the sepsis animal model. In the clinical setting, sepsis is caused by the immune response to a serious infection, most commonly bacteria. In the experimental setting, there are many models of sepsis, such as injection of an exogenous toxin, intestinal leakage, and infusion or instillation of exogenous bacteria. The LPS injection model is simple and has been widely used for sepsis research. We therefore used LPS injection as a sepsis animal model.

Two in-line IV filters are currently in widespread use: 0.2-μm filters for crystalline solutions and 1.2-μm filters for lipid-containing admixtures. Positively charged 0.2-μm filters are able to retain particles, air, microorganisms, and endotoxins [1]. In-line IV filters can remove contaminants from intravenous solutions [19], but may have unwanted effects, such as clogging and a reduced flow rate, drugs binding to the filter itself, and so on. Furthermore, in-line IV filters add cost [19–21]. Some reports have suggested the benefit of in-line IV filters [1, 20, 22, 23], whereas other reports have rejected their routine use [3, 24–26]. To date, the routine use of in-line IV filters is not standard. In addition to these findings, here, we could show new results; polystyrene beads as artificial intravenous fluid contaminants that do not cause embolisms have a potential health hazard in a sepsis animal model. To evaluate the effect of in-line IV filters, further investigation might be required for patients with sepsis.

## Conclusions

We used polystyrene beads as an artificial intravenous fluid contaminant and investigated the effect of injecting polystyrene beads after LPS injection in mice. Damage to glomeruli associated with polystyrene beads only was zero or minimal; however, once the glomeruli had been damaged by LPS, polystyrene beads attached and passed through the renal filtration barrier, accelerated the damage, and led to renal failure.
